# AAV-BR1 does not target endothelial cells in Sprague Dawley rats unlike in mice

**DOI:** 10.17912/micropub.biology.001120

**Published:** 2024-02-29

**Authors:** Ronja Kremer, Anna Williams

**Affiliations:** 1 Institute for Regeneration & Repair, University of Edinburgh, Edinburgh, Scotland, United Kingdom

## Abstract

Adeno-associated viruses (AAVs) are a popular tool in gene therapy approaches and have been engineered to specifically target different cells. There is interest in targeting endothelial cells (ECs) of the blood brain barrier and the AAV2 capsid variant BR1 has been found to transduce ECs with high selectivity in various mice models. However, this has not been tested in rat models. Here, we show that there is no EC transduction with systemic injection of the AAV-BR1-CAG-GFP virus in Sprague-Dawley rats (n=3), but instead transduction of brain parenchymal cells with neuronal morphology. These findings emphasize the importance of species-differences in use of AAVs.

**Figure 1. AAV-BR1-CAG-GFP fails to transduce endothelial cells in Sprague-Dawley rats:**
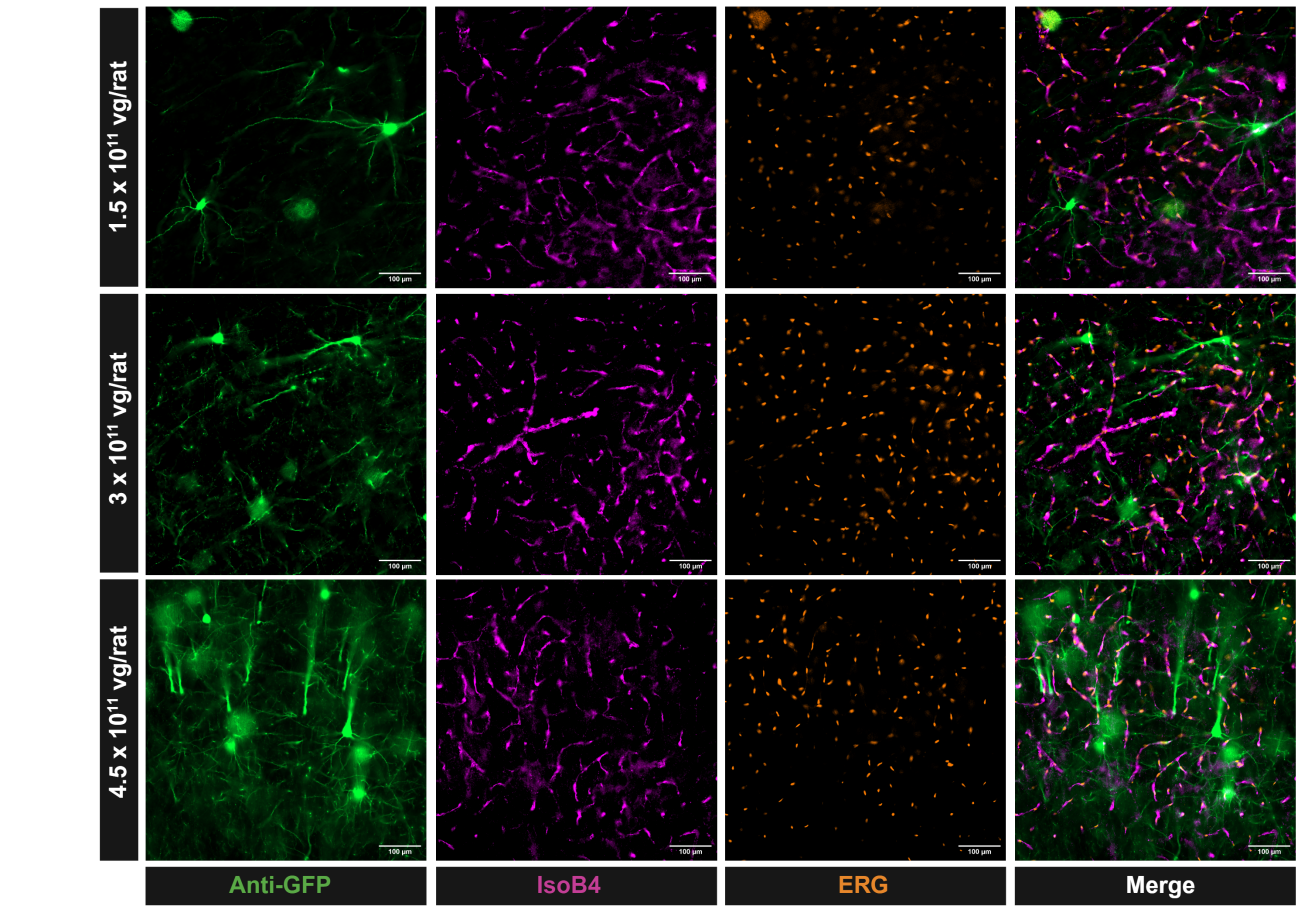
When investigating transduction three weeks after AAV administration, the AAV-BR1-CAG-GFP did not transduce ECs, but instead brain parenchymal cells with the morphology of neurons in young Sprague Dawley rats. Transduction was similar in three different dose trials (1.5 x 10 ^11^ vg/rat, 3 x 10 ^11^ vg/rat, 4.5 x 10 ^11^ vg/rat). Fixed brain tissue was stained for anti-GFP (green), anti-ERG (orange) and IsolectinB4 (IsoB4) (purple).

## Description

Recombinant adeno-associated viruses (rAAVs) have become an increasingly popular tool in gene therapy since their discovery in the mid-1960s (Atchison et al., 1965; Rose et al., 1966) . rAAVs are engineered AAVs, used as transporter tools to transduce cells of interest to deliver their DNA cargo. rAAVs gained their popularity through their easily modifiable structure, their ability to transduce a wide variety of tissues with high selectivity, their high stability after a single administration, and their inability to replicate without a helper virus, making it a safe choice for gene therapy in humans and animal models (Samulski & Muzyczka, 2014).

In our current work, we require an rAAV that targets endothelial cells (ECs) of the blood-brain barrier (BBB) in Sprague-Dawley rats but no other brain cells. When administered systemically, the AAV serotypes AAV9 and AAV2 can transduce both BBB cells and brain parenchymal cells in mice (Dayton et al., 2012; Fu et al., 2003) . Capsid variants, such as the AAV9-derived variant AAV PHP.B, and the AAV2-derived variant AAV-BR1, have been engineered via amino acid insertions to improve BBB transduction in mice (Hordeaux et al., 2018; Körbelin et al., 2016). In particular, the AAV2 capsid variant BR1 transduces ECs in the mouse BBB to a high degree, with only little non-vascular transduction, and has been used in numerous studies using various mouse models (Liu et al., 2019; Nikolakopoulou et al., 2021; Rasmussen et al., 2023; Chao Tan et al., 2019) . However, to our knowledge, there are currently no publications testing the AAV-BR1 variant in rat models.

To test whether AAV-BR1 targets ECs as effectively in rats as it does in mice, we purchased the AAV-BR1-CAG-GFP (SignaGen Laboratories) and administered the AAV via tail injections into three three-week old Sprague-Dawley rats. Due to the size and metabolic differences between mice and rats, we decided to administer three different dosages: One dose that matched the dose frequently given to mice (1.5 x 10 ^11^ vg/rat), one dose that doubled the dose frequently given to mice (3 x 10 ^11^ vg/rat) and one dose that tripled the original mice dose (4.5 x 10 ^11^ vg/rat). The AAV stock solution was diluted in sterile PBS and injected into the tail vein under anaesthesia. Three weeks post-injection, we perfused, harvested, and immunostained the brains for Isolectin B4 (a blood vessel marker), ERG (an endothelial nuclear marker) and GFP (the fluorescence the AAV-BR1 was tagged with).

In Sprague-Dawley rats, GFP expression from the AAV-BR1 was not present in the ECs of any cerebral blood vessels across any brain regions observed, unlike in mice (Liu et al., 2019; C. Tan et al., 2019) . Instead, brain parenchymal cells with the morphology of neurons had high levels of GFP expression, with higher GFP expression in those rats that received higher dosages.

These negative findings in Sprague-Dawley rats highlight the different properties rAAVs can have across species. Körbelin et al. (2016) reported “sporadic” AAV-BR1 neuronal transduction in C57BL/6 mice in their original paper, however, transduction in the Sprague-Dawley rat was exclusive to brain parenchymal cells with neuronal morphology and widespread across brain sections. The observed species-variability may relate to differences in BBB properties between rats and mice as it appears that the AAV-BR1 can pass through the BBB more in Sprague-Dawley rats than in previously tested mice. Age is not relevant, as mice of a similar young age (3-4 weeks of age) have previously showed high EC targeting in previous AAV-BR1 trials (Dogbevia et al., 2020; Liu et al., 2019) and unlike humans, both mice and rats have fully developed BBBs by the time they are born (Ben-Zvi et al., 2014; Daneman et al., 2010) . Therefore, reporting the species-differences in AAV cellular targeting is important and needs to be considered for translational research that aims at human applications.

## Methods

Animals

We used wildtype Sprague-Dawley rats Crl:SD (originally purchased from Charles River, but bred in house) under Home Office regulations (PPL PP1335335).

AAV Injection & Tissue Preparation

AAV-BR1-CAG-GFP was purchased from SignaGen Laboratories (SL116035) and stored at -80°C. Three male 25-day-old Sprague-Dawley wildtype rats received AAV-BR1-CAG-GFP tail injections under anaesthesia with each 1.5x10^ ^11^ , 3x10^ ^11^ and 4.5x10^ ^11^ vg/animal. The virus was diluted in sterile PBS to achieve a volume of 150µl per injection. 3 weeks post injection, the rats were intracardially perfused, brains were harvested and postfixed in 4% PFA at 4°C for two hours. Vibratome sections (60–100 μm) were prepared on the same day and stored in a 24-well plate in PBS at 4°C.

Immunohistochemistry

Sections were washed 3x with PBS and then kept in blocking solution (10%v/v heat inactivated horse serum (HIHS), 0.5%v/v triton (Fisher) in PBS) for one hour at room temperature. Primary antibodies, diluted in blocking solution, were added (Anti-ERG (1:300), IsolectinB4 (1:500), anti-GFP (1:250)) and sections were incubated at 4°C overnight on a shaker. The next day, sections were washed 3x with PBS. Secondary antibodies (647 Streptavidin, 488 donkey anti-goat, 568 donkey anti-rabbit) diluted 1:1000 in PBS were added and incubated for one hour at room temperature. Sections were washed once with PBS-DAPI (1:1000) and twice with PBS. Sections were mounted with Fluoromount (Southern Biotech). Fluorescent images were taken using a Zeiss Observer wide-field microscope.

## Reagents

**Table:** 

**AAV**	**Purchased From**	**Description**
AAV-CAG-GFP (AAV Serotype BR1)	SignaGen Laboratories (SL116035; Titer: > 1x10 ^13^ VG/mL)	Retrieved from Company website: AAV(BR1)-CAG-GFP is a pre-packaged rAAV in serotype BR1 (with capsid from AAV serotype BR1 and 2xITR from AAV serotype 2) which over-expresses EGFP under CAG (also known as CBA) promoter. CAG promoter is a combination of the cytomegalovirus (CMV) early enhancer element and chicken beta-actin promoter for high levels of gene expression in mammalian expression vectors. Ready to use format.

**Table:** 

**Antibody**	**Animal and Clonality/Conjugate**	**Available From**
Recombinant Anti-ERG	Rabbit monoclonal	Abcam (ab92513)
Griffonia Simplicifolia Lectin I (GSL I) Isolectin B4, Biotinylated	Biotin	2bScientific (B-1205)
FITC Anti-GFP antibody	Goat polyclonal	Abcam (ab6662)
Streptavidin, Alexa Fluor™ 647 conjugate	Alexa Fluor 647	ThermoFisher Scientific (S21374)
Donkey anti-Goat IgG (H+L) Cross-Adsorbed Secondary Antibody, Alexa Fluor™ 488	Donkey polyclonal	ThermoFisher Scientific (A-11055)
Donkey anti-Rabbit IgG (H+L) Highly Cross-Adsorbed Secondary Antibody, Alexa Fluor™ 568	Donkey polyclonal	ThermoFisher Scientific (A10042)
